# Association of *XRCC1* Arg399GIn and *Tp53* Arg72Pro polymorphisms and increased risk of uterine leiomyoma - A case-control study

**DOI:** 10.1590/S1415-475738420140359

**Published:** 2015

**Authors:** Minoo Yaghmaei, Saeedeh Salimi, Lida Namazi, Farzaneh Farajian-Mashhadi

**Affiliations:** 1 Department of Obstetrics and Gynecology, School of Medicine, Shahid Beheshti University of Medical Sciences, Tehran, Iran; 2 Cellular and Molecular Research Center, Zahedan University of Medical Sciences, Zahedan, Iran; 3 Department of Clinical Biochemistry, School of Medicine, Zahedan University of Medical Sciences, Zahedan, Iran; 4 Department of Obstetrics and Gynecology, School of Medicine, Zahedan University of Medical Sciences, Zahedan, Iran; 5 Department of Pharmacology, School of Medicine, Zahedan University of Medical Sciences, Zahedan, Iran

**Keywords:** uterine leiomyoma, Tp53, XRCC1, polymorphism

## Abstract

The aim of present study was to investigate the role of the *X-ray repair cross-complementing protein1* (*XRCC1*) and *Tumor protein p53* (*Tp53*) polymorphisms in Uterine Leiomyoma (UL) susceptibility in southeastern Iran. This case control study was performed on 139 women with UL and 149 age, BMI and ethnicity matched healthy women. All women were genotyped for the *XRCC1* Arg399Gln, *XRCC1* Arg194Trp and *Tp53* Arg72Pro polymorphisms. The frequency of *Tp53* 72 Pro/Pro genotype was significantly higher in UL women compared to controls. The risk of UL was 1.5 fold higher in women with the Pro/Pro genotype (OR, 1.5 [95% CI, 1.1 to 2.1], p = 0.012). Moreover, the frequency of the Pro allele was significantly higher in the UL women. Although the frequency of *XRCC1* Arg399Gln genotypes did not significantly differ between UL and control groups before adjusting for age, there was an association between the *XRCC1* Arg/Gln genotype and UL after adjusting for age (OR, 1.8 [95% CI, 1.1 to 3]). No association was observed between the *XRCC1* Arg194Trp polymorphism and UL. The Pro/Pro genotype of *Tp53* Arg72Pro polymorphism was associated with UL susceptibility. In addition, the *XRCC1* Arg/Gln genotype was associated with increased risk of UL after adjusting for age.

## Introduction

Uterine leiomyoma (UL) is a benign neoplasm of the uterine smooth muscle and is originated from the myometrium. ULs are the most common solid tumors of the uterus and pelvis, afflicting 20–25% of women (Hoffman *et al.*, 2012), and are a common condition with various complications, such as abnormal uterine hemorrhage, pressure on adjacent viscera, and even negative effects on reproduction (Haney, 2000). UL is more prevalent at younger age and its prevalence differs in various ethnic groups; it is higher among black women compared to whites. Late reproductive age, nulliparity, and obesity are other predisposing factors for this complication (Flake *et al.*, 2003). Estrogen and Progesterone are growth promoters of UL and probably exert their effects through growth factors that are elevated in uterine leiomyoma (Parker, 2007). It has been reported that 40-50% of women with UL have abnormalities in somatic chromosomes, and the most common observed chromosomal abnormalities are deletions on chromosome 7, trisomy of chromosome 12, and translocation between chromosomes 12 and 14. In addition, atypical and large leiomyoma commonly accompanied with chromosomal abnormalities and a positive relationship between cytogenetic anomalies and UL location has been reported. Therefore, genetic factors could play an important role in UL susceptibility (Medikare *et al.*, 2011; Parker, 2007).

Since ULs are monoclonal tumors which arise from uninhibited division of one myometrial cells, cell cycle regulation and DNA repair failure may be the initial events in formation of UL (Jeon *et al.*, 2005). Several studies have evaluated the correlation between different polymorphisms in genes encoding cell cycle regulatory proteins and susceptibility to various tumors (Jeon *et al.*, 2005; Salimi *et al.*, 2014a).

Up to now, the *Tumor protein p53* (*Tp53*) gene is considered the best tumor suppressor gene and could be activated in response to several cellular signals leading to cell cycle regulation. When DNA is damaged in a cell, the p53 protein causes apoptosis by cell cycle arrest in G1 phase. The *Tp53* gene is located on chromosome 17 and encodes a 53 kDa protein containing 393 amino acids. The *Tp53* gene has various single nucleotide polymorphisms (SNPs) with probable functional effects. Replacement of Arg (CGC) by Pro (CCC) in the transactivation domain of the p53 protein has been the most studied *Tp53* SNP that could affect tumor suppression activity of this protein (Pietsch *et al.*, 2006; Shu *et al.*, 2007). X-ray repair cross-complementing protein1 (XRCC1) or DNA repair protein XRCC1 has a significant effect on DNA repair. XRCC1 interacts with enzymatic proteins of different stages in DNA strand break repair, such as Poly [ADP-ribose] polymerase 1(PARP-1), Apurinic/apyrimidinic (AP) endonuclease-1, polynucleotide kinase, DNA polymerase-b, and DNA ligase IIIa (Caldecott, 2003). Therefore, *XRCC1* polymorphisms that cause amino acid substitutions may alter DNA strand break repair by affecting XRCC1 interaction with other enzymatic proteins (Caldecott, 2003; Jeon *et al.*, 2005; Shen *et al.*, 1998).

There are limited reports about the association between *Tp53* and *XRCC1* polymorphisms and UL susceptibility, with conflicting results. Therefore we aimed to investigate the association between *Tp53* Arg72Pro, *XRCC1* Arg399Gln and *XRCC1* Arg194Trp polymorphisms and uterine leiomyoma women from southeastern Iran.

## Material and Methods

In this study, 139 women with UL in pre-menopause stage who had undergone myomectomy or hysterectomy in Ali-ibn-Abitaleb Hospital, Zahedan, southeastern Iran, were enrolled during 2011–2013. The participants were selected using the convenient sampling method. In all participants, uterine leiomyoma was confirmed pathologically.

One hundred forty nine healthy women, also in pre-menopause stage, were selected as the control group from women referring for routine yearly check-ups and performing the Pap smear test. Both groups were matched with respect to demographic variables such as age, BMI (Body Mass Index) and ethnicity (Fars or Balouch). The participants in the control group had no evidence of uterine leiomyoma upon sonography or examination. Their Pap smear test was also negative. The exclusion criteria were existence of systemic disease and history of malignancy. All participants were Iranian and gave their informed consent before participating in the study. The protocol of this study was approved by the Ethics Committee of Zahedan University of Medical Sciences and conducted in accordance with the Declaration of Helsiniki.

## Genotype analysis

Genomic DNA was extracted from 2 mL peripheral blood leukocytes using the salting out phenol chloroform method and stored at −20 °C until analysis. Tetra-primer amplification refractory mutation system (ARMS) was performed for detection of *XRCC1* Arg399Gln and Arg194Trp polymorphisms, as previously described by (Salimi *et al.*, 2014b).

The analysis of *Tp53* Arg72Pro polymorphism was carried out using the polymerase chain reaction-restriction fragment length polymorphism (PCR-RFLP) method. The fragment containing Tp53 Arg72Pro SNP was amplified using the following forward and reverse primers: 5′- GTC CCA AGC AAT GGA TGA T −3′ and 5′- CAA AAG CCA AGG AAT ACA CG −3′, respectively (Cheng *et al.*, 2012). The total volume of the PCR mixture was 25 μL and contained 200 ng of genomic DNA, 25 pM of each primer, 0.1 mM of dNTP (Fermentas, Lithuania), 1.5 mM MgCl_2_, 2.5 μL 10x PCR buffer, and 1U of *Taq* polymerase (Fermentas, Lithuania). Amplifications were carried out in a Bio-Rad thermal cycler (Bio. Rad, Hercules, CA, USA) using a thermal profile of initial denaturation at 96 °C for 6 min, followed by 30 cycles at 96 °C for 30 s, annealing at 61 °C for 30 s, primer extension at 72 °C for 60 s, and a final extension step at 72 °C for 6 min. The 551 bp PCR product of the Arg72Pro polymorphism was digested by *Bst*U1 (*Bsh1236I*) restriction enzyme (Fermentas, Lithuania) and was incubated at 37 °C overnight. Post-digestion PCR products were identified by electrophoresis on 2.5% agarose gels. The G (Arg) allele had one *Bst*U1 cleavage site and was digested to 443 and 108 bp fragments; whereas the C (Pro) allele had no cleavage site and produced a single 551 bp fragment only.

## Statistical analysis

Statistical analyses were performed using Statistical Package for Social Sciences (SPSS) version 17 (SPSS Inc., Chicago, IL, USA). The frequency of genotypes and alleles was compared between UL women and controls using the Chi-square or Fisher's exact tests. Student's *t-*test was used for comparison of quantitative variables. A p < 0.05 was considered as statistically significant. The association between SNPs and UL were estimated by calculating the odds ratio (OR) and its 95% confidence interval (CI). Linkage disequilibrium (LD) was analyzed using CubeX software (Kumazaki *et al.*, 2002). The independent effect of each risk polymorphism and haplotypes on UL was assessed by Logistic regression analysis.

## Results

The demographic characteristics of the UL women and the control group are shown in Table 1.

**Table 1:** Clinical and demographic characteristics of the uterine leiomyoma women and control group.

	UL women (n = 139)	Controls (n = 149)	p-value
Age (years)	38.4 ± 9.7	37.3 ± 6.7	NS
Marriage status, n (%)	134 (96.4)	145 (97.3)	NS
BMI (kg/m^2^)	25.0 ± 5.8	25.5 ± 5	NS
Parity(n)	3.4 ± 2.6	3.9 ± 2.3	NS
Age of menarche (years)	13.6 ± 1.1	13.3 ± 1.5	NS
Duration of menses (days)	6.1 ± 1.6	5.6 ± 1.5	NS
Menstrual cycle (days)	28.6 ± 2.2	28.0 ± 2.6	NS
Bleeding, n (%)	82 (59)	5 (3.4)	0.0001
Pain, n (%)	41 (30)	11 (7.4)	0.0001

We found no significant differences in age, BMI, parity and marital status between the two groups. There were also no significant differences in menstrual histories, such as age of menarche, duration of menses, and menstrual cycle among UL women and controls. However, the frequency of bleeding and pain was significantly higher in women with UL (p < 0.0001).

The allelic and genotypic frequencies of the *Tp53* Arg72Pro, *XRCC1* Arg399Gln and *XRCC1* Arg194Trp polymorphisms in UL women and controls are shown in Table 2. All loci conformed to the Hardy-Weinberg equilibrium (p > 0.05). The frequencies of *Tp53* 72Arg/Arg, Arg/Pro and Pro/Pro genotypes were 26, 47 and 27% in the UL women and 36, 48 and 16% in control group, respectively. Although the frequency of the heterozygous Arg/Pro genotype was similar in the two groups, the frequency of the homozygous Pro/Pro genotype was significantly higher in UL women compared to the control group. The risk of leiomyoma was 1.5 fold greater in Pro/Pro genotype women compared to the Arg/Arg genotype (OR, 1.5 [95% CI, 1.1 to 2.1], p = 0.012). Moreover, the frequency of the Pro allele was significantly higher in UL women compared to controls (51% *vs.* 40%, p = 0.012). Although there was no association between the Arg/Gln genotype of the *XRCC1* Arg399Gln polymorphism and UL before adjusting for age, we observed a significant association between this genotype and UL susceptibility after adjusting for age. There was no association between *XRCC1* Arg194Trp polymorphism and UL.

**Table 2:** Genotypic and allelic frequencies of *Tp53* Arg/Pro and *XRCC1* Arg399Gln polymorphisms in uterine leiomyoma women and control group.

	UL women (n = 139)	controls (n = 149)	p value	OR (95% CI)	p value	OR (95% CI)*
*Tp53* (Arg72Pro)						
Arg/Arg, n (%)	36 (26)	53(36)		1		1
Arg/Pro, n (%)	65 (47)	72(48)	0.3	1.3(0.8–2.3)	0.3	1.3(0.8–2.3)
Pro/Pro, n (%)	38(27)	24(16)	0.012	1.5(1.1–2.1)	0.014	1.5(1.1–2.1)
*Allele*						
Arg, n (%)	137(49.3)	178(59.7)		1		
Pro, n (%)	141(50.7)	120(40.3)	0.012	1.5(1.1–2.1)		
*XRCC1* (Arg399Gln)						
Arg/Arg, n (%)	63(45.3)	85(57)		1		1
Arg/Gln, n(%)	60(43.2)	50(34)	0.06	1.6(1–2.7)	0.03	1.8(1.1–3)
Gln/Gln, n (%)	16(11.5)	14(9)	0.3	1.2(0.8–1.8)	0.2	1.3(0.9–1.9)
*Allele*						
Arg, n (%)	186(67)	220(74)		1		
Gln, n (%)	92(33)	78(26)	0.07	1.4(1–2)		
*XRCC1* (Arg394Trp)						
Arg/Arg, n (%)	85(61)	94(63)		1		1
Arg/Trp, n (%)	51(37)	51(34)	0.7	1.1(0.7–1.8)	0.7	1.1(0.7–1.8)
Trp/Trp, n (%)	3(2)	4(3)	0.8	0.9(0.4–2)	0.8	0.9(0.4–1.9)
*Allele*						
Arg, n (%)	221(79.5)	239(80)		1		
Trp, n (%)	57(20.5)	59(20)	0.84	1.1(0.7–1.6)		

* Adjusted for age.

Four haplotypes of the *XRCC1* Arg399Gln and *XRCC1* Arg194Trp polymorphisms with two-alleles of each polymorphism are shown in Table 3. There were no differences in haplotype frequency between UL patients and controls as well. Linkage disequilibrium calculated for *XRCC1* gene polymorphisms (rs1799782 and rs25484) were D’ = 0.58, r^2^ = 0.2 in UL and D′ = 0.55, r^2^ = 0.2 in control women.

**Table 3:** Haplotypes frequency of *XRCC1* Arg399Gln and Arg194Trp polymorphisms in uterine leiomyoma women and control group.

XRCC1 Arg399Gln	XRCC1 Arg194Trp	UL women (n = 139)	controls (n = 149)	p value	OR (95% CI)
Arg	Arg	64%	67.6%		1
Arg	Trp	5.8%	6.8%	0.8	0.9(0.3–2.4)
Gln	Arg	15.8%	12.2%	0.4	1.2(0.8–1.7)
Gln	Trp	14.4%	13.5%	0.7	1(0.8–1.3)

## Discussion

The present study revealed that the Pro/Pro genotype of the *Tp53* Arg72Pro polymorphism was associated with higher risk of uterine leiomyoma compared to the Arg/Arg genotype, and the frequency of the Pro allele was significantly higher in UL women. Although there was no association between the *XRCC1* Arg/Gln genotype and UL before adjusting for age, we found an association between Arg/Gln genotype and UL after adjusting for age. In addition *XRCC1* Arg194Trp was not correlated with UL susceptibility.

Although uterine leiomyomas are frequently seen in the uterus, data about its growth and development have rarely been reported. Compared to other tumors, alteration in various genetic targets may lead to dysregulation of smooth muscle cells leading to the typical phenotype of UL. Other than somatic mutations of genes which can lead to proliferation and apoptosis of normal myometrium, the interaction between sexual hormones and growth factors has a crucial role in transforming myometrial smooth muscle cells to leiomyoma (Gittenberger-de Groot *et al.*, 1999; Hirst *et al.*, 2000).

Uterine leiomyoma is pathophysiologically very similar to other fibrotic diseases, including vascular restenosis, atherosclerosis, and interstitial fibrosis that are developed in the kidney, pancreas and liver. It was also proposed that various injuries to the myometrium, including hypoxia, might be important in UL formation (Mesquita *et al.*, 2010).

Considering the role of p53 and XRCC1 in cell cycle and repair regulation, we evaluated the possible effects of *XRCC1* Arg399Gln, *XRCC1* Arg194Trp and *Tp53* Arg72Pro polymorphisms on the susceptibility of UL. There are several studies with controversial results about the association between *Tp53* gene polymorphisms and UL, however in the majority of the studies there was no correlation between *Tp53* Arg72Pro polymorphism and UL susceptibility.

Previous results (Hall *et al.*, 1997) showed that although 6 out of 23 patients with leiomyosarcomas exhibited *Tp53* abnormalities, no patient with leiomyoma had a *Tp53* abnormality. Moreover when Patrakis *et al.* (2003) analyzed exons 5-8 of the *Tp53* gene in UL women, they concluded that the dysregulation of this gene is not required for leiomyoma development. Hsieh *et al.* (2004) showed that the *Tp53* Arg72Pro polymorphism is not a risk factor for UL in Taiwanese women. Similar to the results of our study, carriage of the *Tp53* codon72 polymorphism could be associated with UL susceptibility in a Caucasian population (Germany) and may contribute to the pathology of leiomyoma (Denschlag *et al.*, 2005). In another study on several polymorphisms in the promoter region of the *Tp53* gene, a −216C and a −103G SNP were found to be associated with the development of UL (Hsieh *et al.*, 2007).

There are a few studies about the association between the *XRCC1* Arg399Gln and *XRCC1* Arg194Trp polymorphisms and UL. Jeon *et al.* (2005) reported an association between the *XRCC1* 399Gln allele and UL after adjusting for age, parity, menarche age and body mass index in Korean women. They observed that the incidence of UL was 6.8 fold higher in individuals with the Arg/Gln genotype compared to the Arg/Arg genotype (Jeon *et al.*, 2005). Similarly, we found an association between the Arg/Gln genotype and UL after adjusting for age. In addition Yang *et al.* showed the correlation between Arg280His but not Arg399Gln and Arg194Trp polymorphisms and UL in China (Yang *et al.*, 2010) In another study, Hsieh *et al.* did not observe any association between Arg399Gln and UL in Taiwan (Hsieh *et al.*, 2009). There are reports showing that the 399Gln allele might be associated with more DNA adduct and sister chromatid exchange, as well as mutations. Such findings may, at least in theory, be associated with higher risk of malignancy (Duell *et al.*, 2000; Lunn *et al.*, 1999).

The current study suffers from some limitations; for example low sample size which might affect the results, environmental conditions and different ethnic groups living in southeastern Iran. In addition, if it would have been possible to perform this study on both myomatous and intact tissues the results would have been be more conclusive.

In conclusion, our results show that the Pro/Pro genotype and Pro allele of *Tp53* Arg72Pro polymorphism are probable risk factors for uterine leiomyoma. Although no association was seen between the *XRCC1* Arg399Gln polymorphism and uterine leiomyoma susceptibility before adjusting for age, a significant relationship was observed after adjusting for age.
